# *Escherichia coli* MazF Leads to the Simultaneous Selective Synthesis of Both “Death Proteins” and “Survival Proteins”

**DOI:** 10.1371/journal.pgen.1000390

**Published:** 2009-03-13

**Authors:** Shahar Amitai, Ilana Kolodkin-Gal, Mirit Hananya-Meltabashi, Ayelet Sacher, Hanna Engelberg-Kulka

**Affiliations:** 1 Department of Molecular Biology, The Hebrew University-Hadassah Medical School, Jerusalem, Israel; 2 The Maiman Institute for Proteome Research, The George S. Wise Faculty of Life Sciences, Tel Aviv University, Tel Aviv, Israel; Baylor College of Medicine, United States of America

## Abstract

The *Escherichia coli mazEF* module is one of the most thoroughly studied toxin–antitoxin systems. *mazF* encodes a stable toxin, MazF, and *mazE* encodes a labile antitoxin, MazE, which prevents the lethal effect of MazF. MazF is an endoribonuclease that leads to the inhibition of protein synthesis by cleaving mRNAs at ACA sequences. Here, using 2D-gels, we show that in *E. coli*, although MazF induction leads to the inhibition of the synthesis of most proteins, the synthesis of an exclusive group of proteins, mostly smaller than about 20 kDa, is still permitted. We identified some of those small proteins by mass spectrometry. By deleting the genes encoding those proteins from the *E. coli* chromosome, we showed that they were required for the death of most of the cellular population. Under the same experimental conditions, which induce *mazEF*-mediated cell death, other such proteins were found to be required for the survival of a small sub-population of cells. Thus, MazF appears to be a regulator that induces downstream pathways leading to death of most of the population and the continued survival of a small sub-population, which will likely become the nucleus of a new population when growth conditions become less stressful.

## Author Summary

The enteric bacterium *E. coli*, as most other bacteria, carries a pair of genes on its chromosome; one of them specifies a toxin and the other one an antitoxin. Previously, we have shown that that the *mazEF* toxin–antitoxin system in *E. coli* is responsible for bacterial cell death under stressful conditions. Clearly, a system that causes any given cell to die is not advantageous to that particular cell. On the other hand, the death of an individual cell may be advantageous for the bacterial population as a whole. Here, for the first time, we report that MazF activates a complex network of proteins. Moreover, we also show, for the first time, that MazF affects two opposite processes: cell death and cell survival. We suggest that this dual effect may provide an evolutionary rational for *mazEF*-mediated cell death. When exposed to stressful conditions, most of the cell population undergoes programmed cell death; however, there appears to be an active process that keeps a small fraction of the population alive. When growth conditions become less stressful, it is probably this small sub-population of survivors that becomes the basis of a new cell population.

## Introduction

Toxin-antitoxin modules consist of pairs of genes on the bacterial chromosome [1]–[5]: the downstream gene encodes a stable toxin which causes cell death and the upstream gene encodes a labile antitoxin which counteracts the activity of the toxin. In the *E. coli* chromosome, seven toxin-antitoxin modules have been identified [3], [6]–[10]. Among these, one of the most studied is the *mazEF* system, which was the first to be described as regulatable and responsible for bacterial programmed cell death [11]. *mazF* encodes the stable toxin MazF and *mazE* encodes for the labile antitoxin MazE. MazE is degraded by the ATP-dependent ClpAP serine protease [11]. MazF is an endoribonuclease which cleaves mRNAs at ACA sequences in a ribosome-independent manner [12],[13]. As long as MazE and MazF are co-expressed, MazE counteracts the toxic activity of MazF [11]. Under stressful conditions [11], [14]–[17] that inhibit *mazEF* expression, the *de novo* synthesis of both MazE and MazF is prevented: because MazE is much more labile than MazF, the cellular amount of MazE decreases faster than that of MazF, permitting MazF to act freely, eventually causing cell death [11]. Note that *mazEF*-mediated cell death is a population phenomenon requiring a quorum-sensing factor called EDF [18],[19].

Here, we found that the process of *mazEF*-mediated cell death is more complex than has previously been understood. We show that, as previously reported [12],[20], MazF induction causes the inhibition of protein synthesis. But we were particularly interested to find that this inhibition was not complete: though MazF led to the inhibition of the synthesis of most proteins, it selectively enabled the synthesis of other specific proteins. Some of those specific proteins were required for the death of most of the population. Surprisingly, we also found that MazF enabled the synthesis of proteins that permitted the survival of a small sub-population under those stressful conditions that cause *mazEF*-mediated cell death for the majority of the population. These findings further support our understanding that *mazEF*-mediated cell death is a population phenomenon.

## Results

### Specific Proteins Can Be Synthesized after MazF Induction in *E. coli*

**Figure 1:**
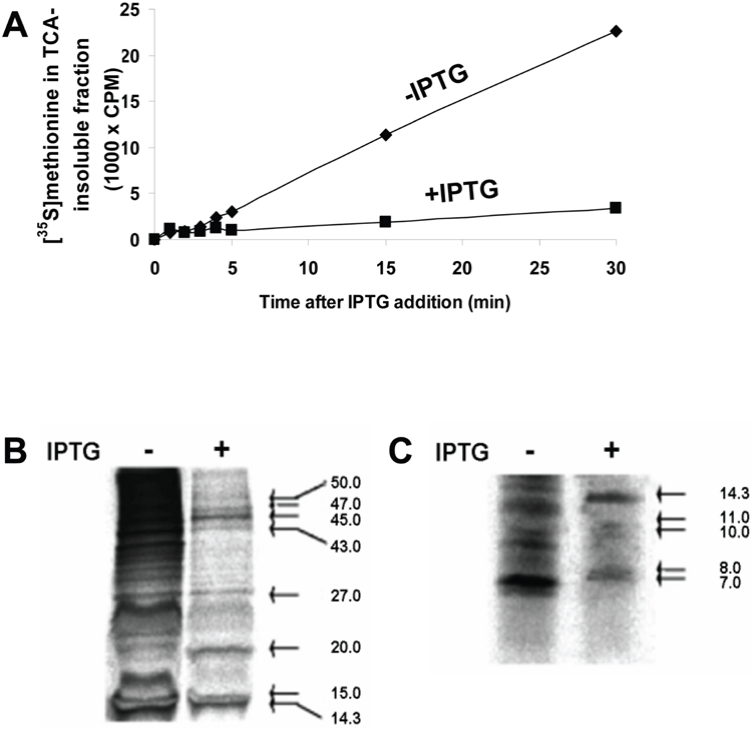
MazF overproduction in *E. coli* does not completely inhibit protein synthesis. *E. coli* strain MC4100 *relA1*, hosting plasmid pSA1 that bears an IPTG-inducible *mazF* gene, was grown to mid-logarithmic phase; the culture was divided into two parts, to one of which IPTG was added. Subsequently, [^35^S]methionine was added to both the induced and the uninduced cultures. (A) At various times, samples were taken from each culture, and the incorporation of radioactive material into the TCA insoluble fraction was determined. In a separate experiment, MC4100 *relA1*/pSA1 was grown to mid-logarithmic phase and further incubated for 15 minutes in the presence or absence of IPTG. Then, the cells were labeled for 5 minutes with [^35^S]methionine, lysed, and (B) run on a 1D-gel for high molecular weight proteins or (C) run on a 1D-gel for low molecular weight proteins. The arrows in B–C indicate the estimated molecular weights (kDa) of proteins synthesized after MazF induction.

It has been previously reported that MazF inhibits protein synthesis [12],[20]. Here, we performed similar studies on the effect of MazF on protein synthesis. We compared the rate of incorporation of [^35^S]methionine into the acid insoluble fraction in MazF-induced and uninduced bacterial cell cultures. Our careful analysis revealed that, after MazF-induction, though most protein synthesis was inhibited, a low level of protein synthesis remained. Even as long as 30 minutes after MazF induction, about 10% protein synthesis was observed compared to the level in the control culture (Figure 1A).

We asked: Was the synthesis of all of the proteins reduced to a basal level? Or perhaps a small selected group of proteins continued to be synthesized? Using 1D-gels, we analyzed the mobility of the proteins that were synthesized after MazF induction: within fifteen minutes, while most protein synthesis was prevented, some clear, sharp radioactive bands appeared (Figure 1B and 1C). These results suggest that while MazF-induction lead to the inhibition of synthesis of most proteins in *E. coli*, the synthesis of an exclusive group of proteins was still permitted. It should be noted that the results shown in Figure 1 were obtained by MazF induction in *E. coli* strain MC4100 *relA1*. This because in our previous studies we have shown that MazF induction causes an irreversible loss of viability in this strain [21]. In addition, we also used 1D-gels to examine the effect of MazF induction on *E. coli* strain MC4100 *relA^+^*. We found that MazF induction affected both strains identically (data not shown).

### MazF Changes the Profile of Protein Synthesis in *E. coli*

**Figure 2:**
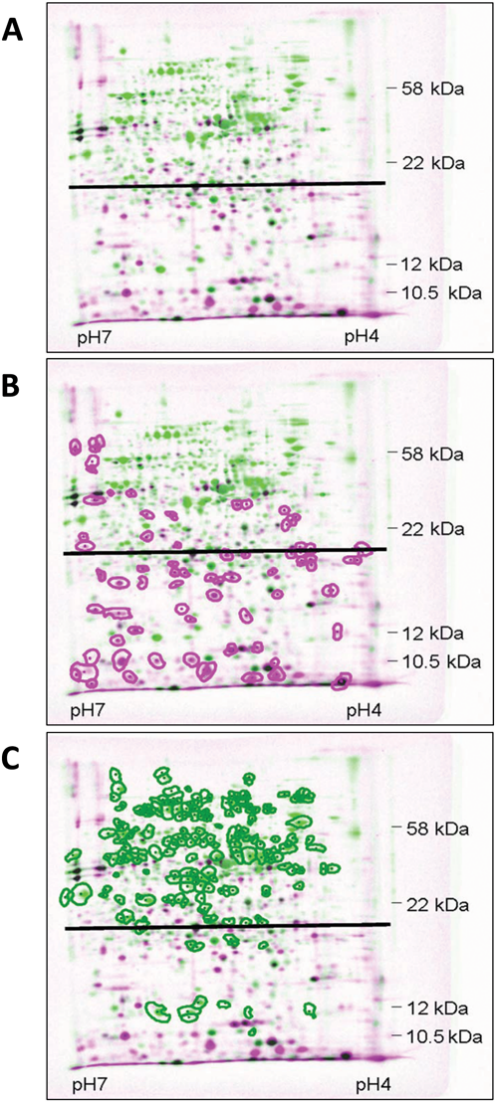
2D-gels showing that MazF induction resulted in a global change in the pattern of protein synthesis. Samples of MC4100 *relA1*/pSA1 cultures, which were grown and treated as described in the legends for Figure 1B and 1C, were also run on 2D-gels. Autoradiograms of these gels were computer stained: magenta for cultures in which MazF had been induced, and green for cultures in which MazF was not induced. (A) The two autoradiograms were superimposed to give a dual-channel image. Consequently, proteins whose level of synthesis was increased after *mazF* induction appear in magenta and those whose level of synthesis was reduced appear in green. Proteins whose level of synthesis did not change after *mazF* induction appear in black. For each spot, the ratio of the results for the MazF-induced and MazF-uninduced cultures was calculated. (B) A copy of (A) in which the spots whose ratio was more than 2 are circled in magenta. (C) A copy of (A) in which the spots whose ratio was less than 0.5 are circled in green. The horizontal black line in Figure 1A–1C indicates a MW of ∼20 kDa.

To better resolve the differences between the profiles of protein synthesis in cultures in which MazF had been induced or not, we took samples which we had previously applied to 1D-gels (Figure 1B and 1C), and subsequently applied them to 2D-gels. Superimposing the autoradiograms of gels of these two cultures revealed that the presence of MazF led to a dramatic change in the profile of protein synthesis in *E. coli* (Figure 2A). This change is reflected in the size of the synthesized proteins. Clearly, the synthesis of proteins whose molecular weight was greater than ∼20 kDa tended to be inhibited (Figure 2A), while the synthesis of proteins whose molecular weight was less than ∼20 kDa tended to be increased. We verified this observation by computer analysis (Figure 2B and 2C): the molecular weights of most of the proteins whose level of synthesis was increased by two times were less than ∼20 kDa (Figure 2B); the molecular weights of most of the proteins whose level of synthesis was decreased by two times were more than ∼20 kDa (Figure 2C). To exclude the possibility that the observed increase in the level of small proteins was a result of degradation of larger proteins, we performed a pulse-chase experiment. During the period examined after MazF induction, we found no change in the general stability of the cellular proteins (Figure S1).

### Some of the Proteins That Are Selectively Synthesized after MazF Induction are Involved in Cell Viability

**Figure 3:**
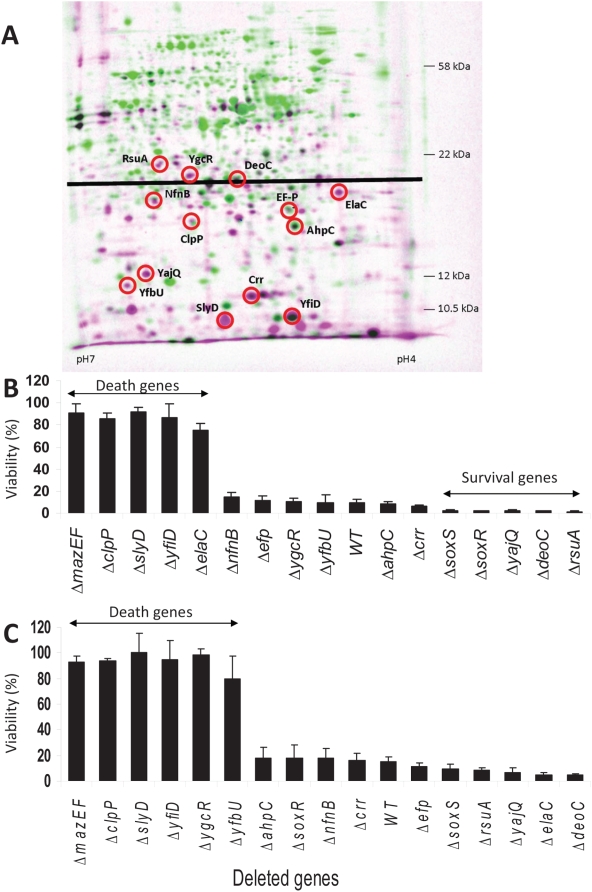
Some *E. coli* genes encoding proteins synthesized after MazF induction are involved in cell death, and some in cell survival. (A) Identified proteins (circled), which are synthesized after MazF induction. (B) Genes encoding the proteins identified in (A) were individually deleted from the chromosome of *E. coli* MC4100 *relA^+^*. These deletion mutants, as well as the WT and its Δ*mazEF* derivative as controls, were briefly treated with spectinomycin to inhibit translation for only a short period, and then plated to determine viability (Materials and Methods). (C) As In (B), but the cells were treated with nalidixic acid to cause damage to the DNA. Genes whose deletion resulted in increased viability were called “Death Genes”. Genes whose deletion resulted in reduced viability were called “Survival Genes.”

We wondered if proteins whose level of synthesis was not reduced after MazF induction were required for cell death. From our 2D-gels (Figure 2), we chose to examine 13 proteins that correspond to this criterion. We extracted these selected proteins from a 2D-gel of an unlabeled MazF-induced culture that we had prepared in parallel with the labeled culture. We identified the nature of those proteins by mass-spectrometry; their positions in the gel are shown in Figure 3A. The identified proteins whose synthesis was increased after MazF induction were: ClpP, Crr, ElaC, NfnB, RsuA, SlyD, YajQ, and YfbU (see Table 1 for the increment in the level of synthesis of each protein). The proteins whose level of synthesis did not change significantly after MazF induction were: AhpC, DeoC, EF-P, YfiD, and YgcR (see Table 1 for the level of synthesis of each protein). To examine the involvement in cell death of each of these proteins, we deleted each of the genes encoding them individually from the *E. coli* MC4100 *relA* ^+^ chromosome.Under stressful conditions, we compared the viability of these deleted mutants to that of the WT and its Δ*mazEF* derivative. We chose stressful conditions that we had previously shown to cause *mazEF*-dependent cell death [14],[17]: (a) brief inhibition of translation by spectinomycin or (b) DNA damage caused by nalidixic acid. As mentioned above, the effect of MazF induction on protein synthesis was identical in both strains *E. coli* MC4100 *relA1* and *E. coli* MC4100 *relA* ^+^. Because *mazEF*-mediated cell death under stressful growth conditions requires the presence of the *relA* gene [17], we only examined the effect of the deleted mutants in strain *E. coli* MC4100 *relA* ^+^.

**Table 1:** Identified proteins selectively synthesized after MazF induction: level of increment in synthesis and their possible role in MazF-downstream pathways.

Protein	Level of increment in synthesis^a^	Role in MazF-downstream pathway induced by inhibition of translation^b^	Role in MazF-downstream pathway induced by DNA damage^c^	Possible function in cell death
**ClpP**	X 2.5	Death	Death	1. MazE degradation 2. EDF generation 3. Additional unknown functions
**SlyD**	X 2.5	Death	Death	Cell permabilization
**YfiD**	X 1.5	Death	Death	Protection of radical-sensitive death proteins
**ElaC**	X 2.5	Death	Irrelevant	RNA processing
**YgcR**	X 1.7	Irrelevant	Death	Unknown
**YfbU**	X 2.1	Irrelevant	Death	Unknown
**YajQ**	X 3.3	Survival	Irrelevant	ROS detoxification
**RsuA**	X 2.1	Survival	Irrelevant	ROS detoxification
**DeoC**	X 0.9	Survival	Irrelevant	1. Catabolism of deoxyribonucleotides 2. ROS detoxification
**NfnB**	X 2.7	Irrelevant	Irrelevant	Irrelevant
**Crr**	X 2.1	Irrelevant	Irrelevant	Irrelevant
**EF-P**	X 0.95	Irrelevant	Irrelevant	Irrelevant
**AhpC**	X 0.76	Irrelevant	Irrelevant	Irrelevant

a The level of increment in protein synthesis was determined as described in Materials and Methods.

b,c The involvement of each protein in *mazEF*-mediated cell death was determined as described in Figure 3 legend.

With respect to cell survival under the stressful conditions that we used, we found three types of mutants. The mutants in the first group behaved like the Δ*mazEF* derivative, that is, most of the population survived. The second group surprised us because the mutants in this group were significantly less viable than was the WT strain. The mutants in the third group behaved like the WT strain and thus, at least under the stressful conditions examined, were irrelevant to our study of cell death.

The mutants in the first group were Δ*clpP*, Δ*slyD*, Δ*yfiD*, Δ*elaC*, Δ*ygcR*, and Δ*yfbU*. Among these, only Δ*clpP*, Δ*slyD*, and Δ*yfiD* behaved like the Δ*mazEF* derivative under both stressful conditions, inhibition of translation (Figure 3B) and DNA damage (Figure 3C). The mutants, Δ*ygcR* and Δ*yfbU* behaved like Δ*mazEF* only under conditions causing DNA damage (Figure 3C). The mutant Δ*elaC* behaved like Δ*mazEF* only under conditions causing the inhibition of translation (Figure 3B). We called *clpP*, *slyD*, *yfiD*, *elaC*, *ygcR* and *yfbU* “Death Genes”, noting that some were involved in cell death under both conditions of inhibition of translation and DNA damage, and some were involved only when the DNA was damaged or when translation was inhibited.

When translation was inhibited only briefly, the mutants in the second group, Δ*yajQ*, Δ*deoC*, and Δ*rsuA*, were significantly less viable than the WT strain (Figure 3B; for a logarithmic-scale view of the results see Figure S2). When we deleted each of these genes individually, the level of survivors in the population was dramatically reduced from about 10% (for the WT strain) to about 2% (for the deleted mutants). Thus, we called *yajQ*, *deoC*, and *rsuA* “Survival Genes”.

Note that we observed no correlation between the growth rates of these mutants and their relevance to *mazEF*-mediated cell death (Figure S3). Here are some examples: (i) on one hand, the growth rates of the mutants of *elaC* and *slyD*, which encode “Death Genes”, resembled that of the Δ*mazEF* strain. On the other hand, the growth rates of the mutants of *yfiD*, *yfbU* and *clpP*, which also encode “Death Genes”, were much slower than the growth rate of the WT strain (Figure S3A); (ii) the growth rate of the mutant of *yfiD*, which encodes a “Death Gene”, resembled that of the mutant of *ahpC*, which did not show any relevance to *mazEF*-mediated cell death (Figure S3A); (iii) the growth rates of any one of the mutants of the genes *rsuA*, *yajQ* or *deoC*, which encode “Survival Genes”, were much slower than the growth rate of the WT strain (Figure S3B). However, the growth rate of the mutant of *efp*, which appeared not to be involved in *mazEF*-mediated cell death, was much slower than the growth rates of those “Survival Genes”. In addition, we compared the CFUs of the above mentioned mutants to the CFUs of the WT and the Δ*mazEF* strains. The CFU was determined at OD_600_ 0.6, the stage where we examined the viability of each strain as shown in Figure 3B and 3C. We did not observe any significant difference between the CFUs of those strains (data not shown).

### *soxS* and *soxR*, Which Are Involved in ROS Detoxification, Are also Involved in *mazEF*-Mediated Cell Death

We have recently discovered that there are two *mazEF*-mediated cell death pathways - an ROS-dependent and ROS-independent [22]. The ROS-dependent pathway is induced by the inhibition of transcription and/or translation, and the ROS-independent patheway is induced by DNA damaging agents. Here we have shown that there are additional genes to *mazEF* that are involved in *mazEF*-mediated cell death. Moreover, we have shown that during this death process, some of those genes function as “Survival Genes”. Therefore, we asked whether the genes *soxS* and *soxR*, known to be involved in ROS detoxification [23], might function as “Survival Genes” in the ROS-dependent *mazEF*-mediated cell death pathway. To this end, each of the genes *soxS* and *soxR* were individually deleted from the chromosome of *E. coli* MC4100 *relA* ^+^. Once again, we compared the viability of those deleted mutants to that of the WT and its Δ*mazEF* derivative under the following stressful conditions: (a) brief inhibition of translation by spectinomycin or (b) DNA damage caused by nalidixic acid.

As we expected, the mutants Δ*soxS* and Δ*soxR* were significantly less viable than the WT strain upon a brief inhibition of translation (Figure 3B) – a stressful condition which induces a ROS-dependent *mazEF*-mediated cell death pathway [22]. The level of survivors in the population was dramatically reduced from about 10% (for the WT strain) to about 1–2% (for the deleted mutants). In contrast, the viability of Δ*soxS* and Δ*soxR* strains resembled that of the WT strain when DNA was damaged (Figure 3C) - a stressful condition which induces a ROS-independent *mazEF*-mediated cell death pathway [22]. Therefore, we suggest that *soxS* and *soxR* function indeed as “Survival Genes” in ROS-dependent *mazEF*-mediated cell death pathway.

## Discussion

Until now, it has been understood that MazF causes the complete inhibition of protein synthesis [12],[24]. Here, when we performed incorporation experiments similar to those previously done by others [12],[20], we indeed observed a dramatic reduction in the level of protein synthesis. However, in contrast to previous reports, we found that the inhibition of protein synthesis was incomplete: a basal level of about 10% protein synthesis remained (Figure 1A). Comparing MazF-induced and MazF-uninduced cultures in 1D-gels revealed that this basal level of protein synthesis remaining after MazF induction represented an exclusive group of proteins (Figure 1B and 1C). More thoroughly analyzing those results on 2D-gel revealed that MazF induction led to a clear change in the pattern of protein synthesis (Figure 2). After MazF induction, we observed an increase in the level of synthesis of proteins whose molecular weight was smaller than ∼20 kDa (Figure 2A and 2B), but a decrease in the level of synthesis of proteins whose molecular weight was greater than ∼20 kDa (Figure 2A and 2C).

MazF is an endoribonuclease that cleaves mRNAs at ACA sequences in a ribosome-independent manner [12],[13]. For this research we used mass-spectrometry to identify 13 proteins that were synthesized within a period of 15 minutes after MazF induction (Figure 3A). We observed that each of the mRNA sequences encoding these proteins carried at least one ACA sequence (data not shown). Since the mRNAs of these proteins carry the MazF's target site, how could those proteins be synthesized after MazF induction? A possible explanation is that there is an as yet unknown mechanism that protects those mRNAs from cleavage by MazF, or at least reduces the rate of cleavage in comparison to the other mRNAs in *E. coli*. We are currently searching for such a mechanism that would allow the selective synthesis of those proteins.

We also found that some of the proteins selectively synthesized after MazF induction were required for cell death (Figure 3B and 3C). Thus, while inhibiting bulk protein synthesis (Figure 1), it seems that MazF also enabled the selective synthesis of proteins essential for cell death (Figure 3). The genes encoding the proteins, which are essential for cell death, can be divided into three groups: (a) *ygcR* and *yfbU* are involved in cell death only when triggered by DNA damage (Figure 3C) but not in cell death triggered by the inhibition of translation (Figure 3B); (b) *elaC* is involved in cell death only when triggered by the inhibition of translation (Figure 3B) but not in cell death triggered by DNA damage (Figure 3C); (c) *clpP*, *slyD*, and *yfiD* are involved in cell death triggered by both the inhibition of translation (Figure 3B) and DNA damage (Figure 3C). These results suggest that there may be at least two separate death pathways that may share some common steps.

What are the roles of the genes that were found by us to be required for *mazEF*-mediated cell death in *E. coli*? (i) *slyD* encodes a peptidyl prolyl cis/trans-isomerase [25],[26] which also functions as an *E. coli* chaperone [27],[28]. SlyD is also involved in the insertion of Ni^2+^ during the maturation of hydrogenases [29]. Moreover, SlyD is required for phage φX174-induced cell lysis [25],[30] where it appears to stabilize the φX174 lysis protein E [27]. We have not yet tested if these functions of SlyD also contribute to *mazEF*-mediated cell death. However, the involvement of SlyD in cell lysis is very intriguing and is currently under our investigation. (ii) *yfiD* encodes a glycyl radical protein that can replace a pyruvate formate-lyase subunit that has been damaged by oxidation [31]. Our recent discovery that ROS is produced during *mazEF*-mediated cell death [22] may provide a clue how the product of *yfiD* is involved: YfiD may enable the ROS-sensitive protein pyruvate formate-lyase to function during the death process. (iii) *clpP* has already been shown to be involved in *mazEF*-mediated cell death [11]. The ATP-dependent ClpAP serine protease degrades MazE antitoxin. When *mazEF* expression is inhibited by specific stressful conditions, there is no *de novo* synthesis of MazE and MazF. Then, ClpAP degrades MazE and the concentration of MazE is reduced. In the absence of MazE, the stable MazF can act freely and cause cell death. In addition, the ATP-dependent ClpXP protease is involved in the synthesis of the communication signaling peptide EDF which is required for *mazEF*-mediated cell death [19]. Here we show that MazF induction causes an increase in the amount of the intracellular ClpP. This may be a part of a positive feedback loop in which the increase in ClpP will cause both a decrease in the level of MazE and an increase in the level of EDF. However, we cannot exclude the possibility that ClpP has an additional role in the cell death process, downstream from MazF activity. (iv) Generally, CCA is the consensus sequence required for a tRNA to be charged with an aminoacyl group. *elaC* encodes RNase BN that cleaves the 3′-terminal portion of tRNA if it differs from CCA [32]. In fact, in *E. coli*, the contribution of RNase BN as a 3′-terminal nuclease remains elusive since *E. coli* has no tRNAs lacking the CCA sequence at their 3′-termini [32],[33]. Recently, it has been suggested that RNase BN may also be responsible for cleaving unstructured RNAs [34]. At this stage we cannot determine whether these functions of RNase BN are connected to *mazEF*-mediated cell death or whether this enzyme may have additional functions essential for *mazEF*-mediated cell death. (v) *yfbU* and *ygcR* encode for proteins of unknown function. Here we show, for the first time, that those genes are required for at least one cellular process in *E. coli* – programmed cell death. The possible roles of the identified “Death Proteins” are summarized in Table 1.

Note that not all of the genes that encoded proteins that were selectively synthesized after MazF induction were part of the death pathway(s). We found that the proteins encoded by *yajQ*, *rsuA*, and *deoC* were not at all involved in the death of the greater part of the cell population. Instead, we found that these genes, whose gene products were selectively synthesized after MazF induction, supported the survival of a small sub-population (Figure 3B). These results indicate that MazF enabled the simultaneous synthesis of specific proteins essential for the death of most of the population and of specific proteins essential for the survival of a small sub-population.

How could these “Survival Genes” contribute to the survival of a small sub-population under stressful conditions causing *mazEF*-mediated cell death? We will discuss each of these genes separately: (i) *soxS* and *soxR* are involved in ROS detoxification [23]. We have recently discovered that there are two *mazEF*-mediated cell death pathways - an ROS-dependent and ROS-independent [22]. The first is induced by the inhibition of transcription and/or translation and the second by DNA damaging agents. Based on our current discovery that *soxS* and *soxR* are essential for the survival of a small sub-population only under inhibition of translation (Figure 3B), we suggest that these genes support cell survival by detoxifying ROS [21]. (ii) *deoC* encodes deoxyribose-phosphate aldolase that is involved in the catabolism of deoxyribonucleosides in *E. coli* [35]. It was reported [36] that strain *E. coli deoC* ^−^, in which a *deoC* of *S. mutans* was expressed, could grow on glucose minimal medium supplemented with deoxynucleotides. This makes it seem likely that the major sub-population, which undergoes a *mazEF*-mediated cell death process, may releases deoxynucleotides into the medium. The rest of the population, still alive, could survive by using those deoxynucleotides as a carbon and energy source. Another possibility is that *deoC* may contribute to the survival of a small sub-population by being involved in ROS detoxification. Like *soxS* and *soxR*, which are known to be involved in ROS detoxification [23], *deoC* is essential to cell survival only upon the inhibition of translation (Figure 3B) which triggers ROS-dependent *mazEF*-mediated cell death [22]. (iii) *rsuA* encodes an enzyme which catalyzes pseudouridylation at position 516 in the 16S rRNA [37],[38], and (iv) *yajQ* encodes a protein of unknown function. We cannot yet determine how *rsuA* and *yajQ* can contribute to the above mentioned survival of a small sub-population. However, as suggested for *deoC*, we can speculate that these genes may also be involved in ROS detoxification. Once again, we base our suggestion on our finding that these genes are involved in cell survival only in ROS-dependent *mazEF*-mediated cell death pathway [22], triggered by the inhibition of translation (Figure 3B), and not in ROS-independent *mazEF*-mediated cell death pathway [22], triggered by DNA damage (Figure 3C). The possible roles of the identified “Survival Proteins” are summarized in Table 1.

Here we have shown, for the first time, that MazF induced downstream pathways required for both death and life, confirming our hypothesis [7],[8],[21] that MazF is a regulator of cell death rather than the cell executioner. This dual effect of MazF on two such opposite processes, cell death and cell survival, may provide an evolutionary rational to *mazEF*-mediated cell death. We suggest that when exposed to stressful conditions, while most of the bacterial cell population undergoes programmed cell death, an active process keeps a small fraction of the population alive. When the growth conditions become less stressful, these survivors probably become the nucleus of a new population. We have previously reported [18],[19] that *mazEF*-mediated cell death is a population phenomenon requiring a quorum-sensing factor called EDF. That *mazEF*-mediated cell death is indeed a population phenomenon is strongly supported by the results of our work here showing that MazF induction contributed both to the death of most of the population and to the survival of a small sub-population. It should be noted that an analogous phenomenon, in which an active process of cell death of a sub-population enables the survival of the rest of the population, was found in *Bacillus subtilis* [39],[40].

**Figure 4:**
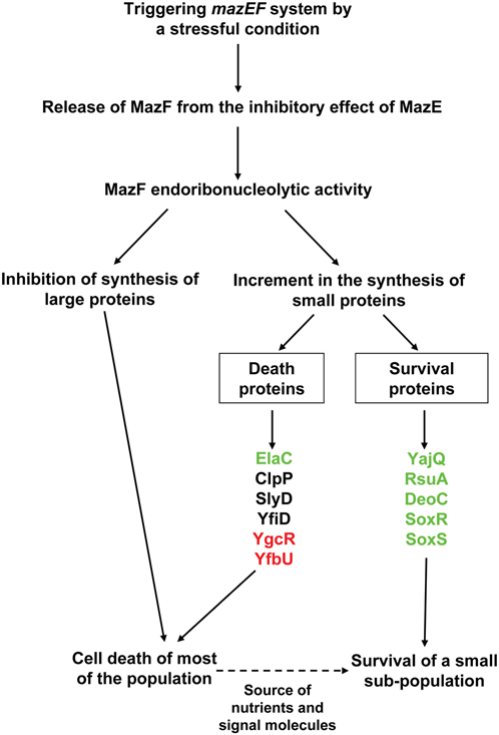
The *mazEF*-mediated cell death process - an extended model. Text and arrows in black represent the *mazEF*-mediated cell death process that occurs following either DNA damage or brief inhibition of protein synthesis. Text in red represents proteins that were found to participate in cell death, only when *mazEF* was induced by DNA damage. Text in green represents proteins that were found to participate in cell death and survival, only when *mazEF* was induced by brief inhibition of translation. For a discussion of this model please see the text.

Based on our present results, we have developed our model [8] for *mazEF*-mediated cell death process (Figure 4). As we have shown previously [11], [14]–[17], inhibiting *mazEF* expression by various stressful conditions leads to the reduction in the cellular amount of the labile antitoxin MazE. Thereby, the stable toxin MazF can act freely as an endoribonuclease. As we have reported here, the unrestricted action of MazF leads to the inhibition of the synthesis of many proteins, particularly those larger than ∼20 kDa (Figure 2). However, some proteins, particularly those smaller than ∼20 kDa, can still be selectively synthesized (Figure 2). At least six of those proteins, which are selectively synthesized after MazF activation, are necessary for implementing the death of most of the cell population (Figure 3). Moreover, it seems that more than one death pathway can be activated by MazF. The specific pathway chosen appears to be a function of the particular stressful condition, like DNA damage or the inhibition of protein synthesis (Figure 3). We believe that the cell is led towards its own death by the combination of the inhibition of the general synthesis of proteins, necessary for life, and the parallel synthesis of proteins necessary for the death process. Furthermore, while at least six of the selectively synthesized proteins are required for the death of most of the cell population, at least three other small proteins, also selectively synthesized after MazF activation, are required for the survival of a small sub-population (Figure 3). It seems likely that the survival of that small sub-population would be supported by the dead cells, that would then release nutrients and other factors, like signal molecules, essential for survival.

## Materials and Methods

### Strains and Plasmids

We used *E. coli* strains MC4100 *relA1*, MC4100 *relA^+^*, and MC4100 *relA^+^* Δ*mazEF*, which we have described previously [11],[14],[15],[17]. In addition, using the procedure of Datsenko and Wanner [41], we constructed the following derivatives of MC4100 *relA^+^*: MC4100 *relA^+^* Δ*ahpC*, MC4100 *relA^+^* Δ*clpP*, MC4100 *relA^+^* Δ*crr*, MC4100 *relA^+^* Δ*deoC*, MC4100 *relA^+^* Δ*efp*, MC4100 *relA^+^* Δ*elaC*, MC4100 *relA^+^* Δ*nfnB*, MC4100 *relA^+^* Δ*rsuA*, MC4100 *relA^+^* Δ*slyD*, MC4100 *relA^+^* Δ*soxS*, MC4100 *relA^+^* Δ*soxR*, MC4100 *relA^+^* Δ*yajQ*, MC4100 *relA^+^* Δ*yfbU*, MC4100 *relA^+^* Δ*yfiD*, and MC4100 *relA^+^* Δ*ygcR*. Plasmid pSA1 is a derivative of pQE30 (Qiagen, Hilden, Germany) bearing *lacI^q^* and also bears *mazF* under the control of the T5 promoter and the *lac* operator.

### Media and Growth Conditions

For viability assays, cells were grown in M9 minimal medium containing 1% glucose and a mixture of amino acids (except for tyrosine and cysteine), each at 100 µg/ml. The cells were plated on rich Luria-Bertani (LB) agar plates as described previously [14],[17]. For labeling experiments, cells were grown in M9 minimal medium containing 0.2% glucose and a mixture of amino acids (except for methionine, tyrosine, tryptophan, and cysteine), each at 20 µg/ml.

### Assay for the Effect of MazF Induction on Protein Synthesis by Measuring Incorporation of [^35^S]methionine into a TCA-Insoluble Fraction

Strain MC4100 *relA1* was transformed with pSA1 bearing *mazF*. The culture was grown in M9 medium without methionine) with the addition of 100 µg/ml ampicillin, at 37°C. When the culture was in mid-logarithmic phase (OD_600_ 0.5), it was divided in half, and each half was diluted 1∶200. Cold methionine at 0.125 µg/ml was added to both sub-cultures. One sub-culture was kept as a control; to the other sub-culture 5 µM isopropyl β-D-thiogalactopyranoside (IPTG) was added to induce MazF synthesis. Immediately after induction by IPTG, both sub-cultures were labeled with [^35^S]methionine (13.75 µCi/ml) and incubated at 37°C, without shaking. At various time intervals, samples were withdrawn and the reactions were stopped by the addition of trichloroacetic acid (TCA) to a final concentration of 5%, after which the reaction tubes were placed in ice. The samples were filtered through 0.45 µM filters using a vacuum pump. A BETAmatic I/II scintillation counter (KONTRON) was used to determine the radioactivity in the TCA-insoluble material.

### 1D-Gel and 2D-Gel Analysis of the Effect of MazF Induction on Protein Synthesis

*E. coli* MC4100 *relA1*, harboring plasmid pSA1, was grown to mid-logarithmic phase (OD_600_ 0.5) as described above. Then, the culture was divided into two and 1 mM IPTG was added to one half of the culture. Both sub-cultures were incubated at 37°C, without shaking, for 15 min. [^35^S]methionine (110 µCi/ml) was added to each sub-culture which were then further incubated at 37°C, without shaking, for 5 min. The labeling reaction was terminated by placing the samples in liquid nitrogen. The samples were centrifuged at 14000 rpm, for 10 min. The pellets were washed in 50 mM tris(hydroxymethyl)aminomethane (Tris) pH 7.5 and then resuspended in lysis buffer (0.5 mg/ml lysozyme, 10 mM Tris pH 8, 1 mM ethylene diamine tetraacetic acid (EDTA), 20 µg/ml DNase, 50 µg/ml RNase) and 10% sodium dodecyl sulfate (SDS). Lysates were incubated at 90°C for 5 min. These prepared lysates were loaded either onto a 10% SDS polyacrylamide gel [42] or onto a 16% *N*-Tris(hydroxymethyl)methylglycine (Tricine)-SDS polyacrylamide gel [43]. In addition, samples prepared for 1D-gel analysis were centrifuged (8000 rpm at 4°C for 5 min) and then washed twice with cold Tris-EDTA and Phenylmethylsulfonyl Fluoride (TE-PMSF) (10 mM Tris pH 7.5, 1 mM EDTA, 1.4 mM PMSF). The washed cells were resuspended in 0.5 ml of TE-PMSF and disrupted by sonication. Cell debris and protein aggregates were removed by centrifugation at 14000 rpm at 4°C for 30 min. The protein concentrations of the remaining supernatants were determined using the Bradford method with the BioRad Protein Assay kit (Hercules,CA, USA) [44]. These protein containing supernatants were lyophilized and further prepared for 2D-gel analysis as described previously [45]. Both the 2D-gel analysis and the determination of the level of increment in protein synthesis were done by the use of Delta2D software (DECODON GmbH, Greifswald, Germany).

### Metabolic Stability of *E.coli* Proteins after MazF Induction

*E. coli* strain MC4100 *relA1* was transformed with pSA1 bearing *mazF*. The culture was grown in M9 medium without methionine with the addition of 100 µg/ml ampicillin at 37°C. When the culture was in mid-logarithmic phase (OD_600_ 0.5), it was labeled with [^35^S]methionine (220 µCi/ml). The labeled culture was incubated at 37°C, without shaking, for 5 min. Then, both cold methionine (2 mg/ml) and 1 mM IPTG were added. The culture was further incubated at 37°C, without shaking. Over a period of 16 min, samples were withdrawn from the culture every 4 min and placed in liquid nitrogen. The samples were centrifuged at 14000 rpm for 10 min. The pellets were washed in 50 mM Tris pH 7.5 and then resuspended in lysis buffer (0.5 mg/ml lysozyme, 10 mM Tris pH 8, 1 mM EDTA, 20 µg/ml DNase, 50 µg/ml RNase) and 10% SDS. Lysates were incubated at 90°C for 5 min. These prepared lysates were loaded onto 10% SDS polyacrylamide gel [42].

### Identifying Proteins Synthesized after the Induction of MazF Synthesis

To identify proteins synthesized after the induction of MazF, we used an autoradiogram of a 2D-gel analysis of a labeled, MazF-induced culture. We chose spots that corresponded to proteins whose level of synthesis was either not changed or even increased after MazF induction (Figure 3A). Those selected proteins were extracted from a parallel 2D-gel of an unlabeled, MazF-induced, culture; the proteins were identified by mass-spectrometry (MALDI-MS) as described previously [45].

### Viability Assays

*E. coli* MC4100 *relA* ^+^ and its derivatives were grown in M9 minimal medium at 37°C. After 12–16 hours of growth, they were diluted 1∶100 in M9 minimal medium and grown again at 37°C. When the cultures reached OD_600_ 0.6, 0.5 ml aliquots were taken from the cultures, put into Eppendorf tubes, and incubated, without shaking, at 37°C. After 10 min of incubation, *mazEF* dependent death was induced by the addition to each sample of either 2 mg/ml spectinomycin or 1 mg/ml nalidixic acid. After an additional 10 min of incubation, without shaking, at 37°C, the samples were centrifuged for at 14000 rpm for 5 min. After centrifugation, the supernatants were removed and the pellets were resuspended in 0.5 ml of pre-warmed saline. The samples were serially diluted in pre-warmed LB and plated on pre-warmed LB plates and incubated at 37°C. The percentage of survival was determined by dividing the number of colonies obtained from the “treated” sample by the number of colonies obtained from the “untreated” sample.

## Supporting Information

Figure S1 MazF induction does not lead to the global degradation of *E. coli* proteins. *E. coli* strain MC4100 *relA1*, hosting plasmid pSA1 that bears an IPTG-inducible *mazF* gene, was grown to mid-logarithmic phase. Then, the culture was labeled with [^35^S]methionine for 5 min. Subsequently, both cold methionine and IPTG were added to the culture (time zero). At the indicated time points, samples were taken from the culture, lysed, and run on a 1D-gel for high molecular weight proteins.
(1.97 MB EPS)

Figure S2 A logarithmic-scale view of the differences in the viability under various stressful conditions of WT and “Survival Gene” mutants. To emphasize the difference between the WT and the deletion mutant strains, the results presented in Figure 3B and 3C for the WT, Δ*soxS*, Δ*soxR*, Δ*yajQ*, Δ*deoC*, and Δ*rsuA* strains, are shown here in logarithmic scale.
(9.62 MB EPS)

Figure S3 How did deleting genes encoding proteins that were synthesized after MazF induction affect growth rate? The strains whose viability was examined in Figure 3B and 3C were grown, as described in Materials and Methods, until the stationary phase. The optical density of each strain was measured at the indicated time points. (A) A comparison between the growth rates of the mutants of “Death Genes” and the ones of WT and Δ*mazEF* strains. (B) A comparison between the growth rates of the mutants of “Survival Genes” and the ones of WT and Δ*mazEF* strains. Growth rates of the mutants of the genes, which showed no relevance to *mazEF*-mediated cell death, are distributed between (A) and (B).
(0.59 MB EPS)
